# Causal Associations between Paternal Longevity and Risks of Cardiovascular Diseases

**DOI:** 10.3390/jcdd9080233

**Published:** 2022-07-26

**Authors:** Mengjin Hu, Xiaoning Wang, Jiangshan Tan, Jingang Yang, Xiaojin Gao, Yuejin Yang

**Affiliations:** 1 State Key Laboratory of Cardiovascular Disease, Fuwai Hospital, National Center for Cardiovascular Diseases, Chinese Academy of Medical Sciences & Peking Union Medical College, Beijing 100037, China; 18393911603@163.com (M.H.); happyshown@163.com (J.T.); yangjingang@fuwai.com (J.Y.); 2 School of Medicine, Shandong University, Jinan 250012, China; 18769742156@163.com

**Keywords:** paternal longevity, coronary heart disease, peripheral artery disease, cardiovascular diseases, Mendelian randomization

## Abstract

Background: Observational studies have suggested that paternal longevity is associated with reduced risks of cardiovascular diseases, yet the causal association remains to be determined. Objectives: To investigate whether Mendelian randomization (MR) results support a causal role of paternal longevity for risks of cardiovascular diseases. Methods: Genetic variants associated with paternal longevity and cardiovascular diseases were obtained from public genome-wide association study data. We used inverse variance weighted MR under a random-effects model to provide causal estimates between paternal longevity and cardiovascular diseases. Results: Paternal longevity was associated with decreased risks of coronary heart disease (odds ratio (OR): 0.08; 95% confidence interval (CI): 0.02–0.37; *p* = 0.001) and peripheral artery disease (OR: 0.15; 95% CI: 0.03–0.65; *p* = 0.011). No significant differences were observed in hypertension, atrial fibrillation, heart failure, transient ischemic attack, ischemic stroke, or cardiac death. The weighted median method revealed consistent results between genetically instrumented paternal longevity and decreased risk of coronary heart disease and peripheral artery disease. No significant differences were observed in the MR-Egger results. Multivariable MR consistently indicated causal associations between paternal longevity and decreased cardiovascular diseases. The leave-one-out analysis suggested that the causal associations were not affected by individual single-nucleotide polymorphisms. The intercept of the MR-Egger estimator and funnel plot revealed no indication of horizontal pleiotropic effects. Conclusions: Our MR analyses supported a causal role of paternal longevity for decreased risks of coronary heart disease and peripheral artery disease, which highlighted the need for better monitoring and intervention of cardiovascular diseases in populations with premature paternal death.

## 1. Introduction

A family history of coronary heart disease, especially premature coronary heart disease, is a well-established risk factor for coronary heart disease in the offspring [1]. However, less attention has been paid to the effect of parental longevity on cardiovascular diseases in the offspring. Longevity is influenced by the living environment and genes coding for survival. If environmental factors remained constant from one generation to another, there may be a similarity in longevity between parents and offspring. Concordant to the assumption, offspring of centenarians were more likely to survive to 100 years than their matched controls who had a parent who died during average life expectancy [2]. Moreover, parental survival was found to be an independent predictor of longevity in middle-aged persons [3].

In the pioneering research published in 1971, the death rates attributed to coronary heart disease, hypertension, and stroke were significantly lower among populations with long-lived parents compared to populations with short-lived parents [4]. Studies of twins also indicated that death from coronary heart disease at younger ages was influenced by genetic factors [5]. Compared to controls, centenarian offspring demonstrated a delayed age of onset of many age-related morbidities, especially cardiovascular disease and cardiovascular risk factors [2]. In the Primary Prevention Study, the protective effect of paternal, but not maternal, longevity on coronary disease was documented [6]. Similarly, the SUVIMAX Vascular Study also suggested that premature paternal death was associated with higher incidences of carotid atherosclerosis and aortic arterial stiffness in adult offspring, while no differences were observed in populations with premature maternal death [7]. All of the aforementioned studies suggested that parental longevity, particularly paternal longevity, may play a significant role in predicting cardiovascular diseases in offspring. Despite these, however, drawing causal inferences from observational studies can be challenging because of the existence of residual confounding and potential reverse causation. In that case, Mendelian randomization (MR) analysis may be helpful in clarifying the causal associations between paternal longevity and cardiovascular diseases. Using genetic variants as instruments for a modifiable exposure, MR analysis is able to investigate causal relationships between risk factors and diseases, as genetic variants are randomly allocated at birth and therefore independent of confounding factors and reverse causation associated with observational studies.

Applying a two-sample MR analysis, we sought to investigate the causal role of paternal longevity for risks of cardiovascular diseases, including coronary heart disease, hypertension, atrial fibrillation, heart failure, transient ischemic attack, ischemic stroke, peripheral artery diseases, and cardiac death. Moreover, multivariable MR, a newly developed extension to MR, was also performed to estimate the causal effects of paternal longevity after adjusting for other risk factors [8].

## 2. Methods

### 2.1. Data Sources

The MR analysis used published summary-level data from genome-wide association studies (GWAS) of the interested traits in predominantly European populations. Effect size estimates for single-nucleotide polymorphism (SNP) associated with paternal longevity (*n* = 341,118) were obtained from the UK Biobank study [9]. Genetic data for coronary heart disease (*n* = 184,305) were obtained from the CARDIoGRAMplusC4D Consortium (Coronary Artery Disease Genome-wide Replication and Meta-Analysis) [10]. Genetic data for hypertension (*n* = 218,792), atrial fibrillation (*n* = 127,442), transient ischemic attack (*n* = 214,634), peripheral artery disease (*n* = 218,792), and cardiac death (*n* = 218,792) were obtained from the FinnGen Consortium. Genetic data for heart failure (*n* = 977,323) were obtained from the HERMES Consortium (Heart Failure Molecular Epidemiology for Therapeutic Targets) [11]. Genetic data for ischemic stroke (*n* = 440,328) were obtained from the results conducted by Malik et al. [12]. Details of included GWAS are provided in Supplementary Materials Table S1. Ethical approval was not required for the present MR analysis, as all analyses were based on public GWAS data, which had been approved by the corresponding ethical review board.

### 2.2. SNP Selection

We selected independent SNPs strongly associated with paternal longevity (*p* < 5 × 10^−8^). The pairwise-linkage disequilibrium was used to evaluate the independence among selected SNPs [13]. When r^2^ > 0.001 (clumping window 10,000 kb), we deleted the SNP correlated with more SNPs or with a higher *p* value. To assess the strength of SNPs, F-statistics were calculated, with F ≥ 10 being deemed a strong instrument.

### 2.3. Statistical Analysis

The MR analysis contains three key assumptions: (1) genetic variant is significantly associated with exposure, (2) genetic variant is independent of any confounding factors of exposure and outcome, and (3) genetic variant affects outcome entirely through exposure. The primary analysis was inverse variance weighted (IVW) MR under a random-effect model, which assumed all genetic variants as valid instruments. Weighted median and MR-Egger methods were also performed [14,15]. The weighted median method can reveal consistent estimates even if up to 50% of the weight in the MR analysis comes from invalid genetic variants [16]. The MR-Egger method can test whether the IVW estimates are biased by unbalanced horizontal pleiotropic effects. The intercept from MR-Egger indicates the extent of directional pleiotropy [17]. In addition, multivariable MR analyses were also performed, adjusting for smoking, body mass index, systolic blood pressure, and total cholesterol [8]. A leave-one-out analysis was used to examine whether the associations examined were influenced by individual SNP. Cochrane’s Q value was used to assess the heterogeneity among selected SNPs. Results were reported as odds ratio (OR) with the corresponding 95% confidence interval (CI). To account for multiple testing in paternal longevity with eight outcomes, a Bonferroni-corrected threshold of *p* < 6.25 × 10^−3^ (α = 0.05/8 outcomes) was used. *p* values between 6.25 × 10^−3^ and 0.05 were considered suggestive evidence of associations, and further confirmation was required. All statistical analyses were performed using the “TwoSampleMR” packages in R version 4.0.3 (R Foundation for Statistical Computing, Vienna, Austria).

## 3. Results

### 3.1. Selected SNPs

The baseline characteristics of included GWAS data are shown in Supplementary Materials Table S1. The included GWAS were published between 2015 and 2021 and were mainly based on the European population. Eventually, twelve SNPs were included in the MR analysis as instrumental variables for paternal longevity, including rs59660701, rs13070730, rs11709525, rs186696265, rs4714070, rs2179517, rs1556516, rs10774625, rs2071382, rs56390833, rs429358, and rs12461964 (Table 1).

### 3.2. Associations with Cardiovascular Diseases

**Figure 1:**
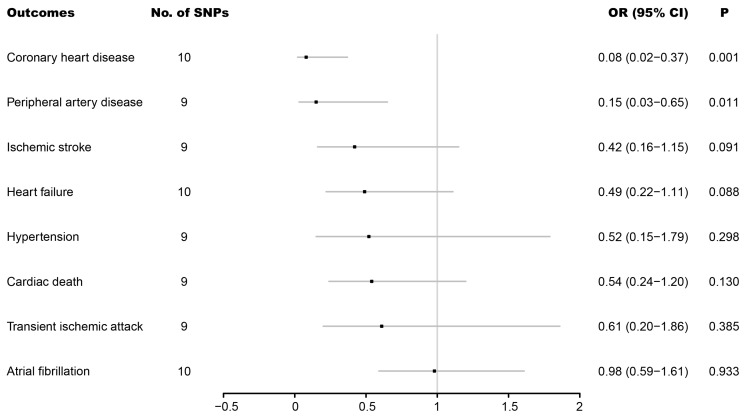
Associations of genetically predicted father’s age at death with eight cardiovascular diseases.

As shown in Figure 1, genetically instrumented paternal longevity was associated with a decreased risk of coronary heart disease (OR: 0.08; 95% CI: 0.02–0.37; *p* = 0.001). Suggestive evidence between genetically instrumented paternal longevity and a decreased risk of peripheral artery disease (OR: 0.15; 95% CI: 0.03–0.65; *p* = 0.011) was also found. However, no significant differences were observed in ischemic stroke (OR: 0.42; 95% CI: 0.16–1.15; *p* = 0.091), heart failure (OR: 0.49; 95% CI: 0.22–1.11; *p* = 0.088), hypertension (OR: 0.52; 95% CI: 0.15–1.79; *p* = 0.298), cardiac death (OR: 0.54; 95% CI: 0.24–1.20; *p* = 0.130), transient ischemic attack heart failure (OR: 0.61; 95% CI: 0.20–1.86; *p* = 0.385), or atrial fibrillation (OR: 0.98; 95% CI: 0.59–1.61; *p* = 0.933. CI, confidence interval; OR, odds ratio; SNP, single-nucleotide polymorphism.

The weighted median results in Table 2 revealed consistent results between genetically instrumented paternal longevity and decreased risk of coronary heart disease (OR: 0.12; 95% CI: 0.05–0.30; *p* < 0.001) and peripheral artery disease (OR: 0.12; 95% CI: 0.03–0.43; *p* = 0.001). Additionally, genetically instrumented paternal longevity and decreased risk of heart failure (OR: 0.46; 95% CI: 0.27–0.77; *p* = 0.003) were also found in the weighted median results. However, no significant differences were observed in the MR-Egger results. In multivariable MR analysis, genetically instrumented paternal longevity was associated with decreased risks of coronary heart disease (OR: 0.16; 95% CI: 0.10–0.25; *p* < 0.001), hypertension (OR: 0.54; 95% CI: 0.38–0.76; *p* = 0.001), heart failure (OR: 0.47; 95% CI: 0.34–0.66; *p* < 0.001), and cardiac death (OR: 0.38; 95% CI: 0.21–0.70; *p* = 0.002).

The leave-one-out analysis suggested that the genetically instrumented paternal longevity and decreased risks of coronary heart disease and peripheral artery disease were not driven by individual SNP (Figure S1).

The Cochran’s Q value implied a substantial heterogeneity in the majority of clinical outcomes (Table 3). Therefore, IVW under a random-effects model was adopted to mitigate the influence of heterogeneity. The test of intercept of the MR-Egger estimator showed no significant horizontal pleiotropic effects for the included variants (Table 3), which was in line with the results from the funnel plot in Supplementary Figure S2.

## 4. Discussion

Using genetic information from 12 SNPs with significant association with paternal longevity, we supported a causal role of paternal longevity for cardiovascular diseases, including decreased risks of coronary heart disease and peripheral artery disease without effects on hypertension, atrial fibrillation, heart failure, transient ischemic attack, ischemic stroke, or cardiac death.

The protective effects of paternal longevity on cardiovascular diseases have been demonstrated in observational studies [2,6,18]. However, the causality between these conditions and to what extent paternal longevity may affect cardiovascular diseases remain to be determined. In the prospective Primary Prevention Study, a total of 6242 men aged 51 to 59 years were included and divided into <70, 70–74, 75–79, 80–84, 85–90, and >90 years groups according to father’s age at death. Coronary disease-related death decreased continuously with increasing paternal longevity, with a relative risk (RR) of 0.89 (95% CI: 0.72–1.10), 0.75 (95% CI: 0.61–0.93), 0.68 (95% CI: 0.55–0.84), 0.74 (95% CI: 0.56–0.97), and 0.41 (95% CI: 0.23–0.73), respectively, for the 70–74, 75–79, 80–84, 85–90, and >90 years groups compared with the reference group of <70 years (*p* for trend <0.0001) after adjusting for age and cardiovascular risk factors. Moreover, the cardiovascular death was also decreased continuously with increasing paternal longevity, with an RR of 0.93 (95% CI: 0.77–1.11), 0.81 (95% CI: 0.69–0.97), 0.74 (95% CI: 0.62–0.88), 0.68 (95% CI: 0.54–0.87), and 0.60 (95% CI: 0.40–0.90), respectively, for the 70–74, 75–79, 80–84, 85–90, and >90 years groups compared with the reference group of <70 years (*p* for trend <0.0001). This suggested that paternal longevity may confer additional information on disease risks in the offspring that is not affected by age or cardiovascular risk factors. In the nationwide New England Centenarian Study, the prevalence of coronary heart disease (OR: 0.44, 95% CI: 0.24–0.80), hypertension (OR: 0.34, 95% CI: 0.21–0.55), and diabetes (OR 0.41, 95% CI 0.15–1.12) was significantly lower in the centenarian offspring (*n* = 177) compared with controls (*n* = 166) after multivariate adjustment. No difference was found in the prevalence of arrhythmia [2]. Moreover, centenarian offspring significantly delayed the onset of diabetes, stroke, coronary heart disease, and hypertension by 9.0 (*p* = 0.002), 9.0 (*p* = 0.02), 5.0 (*p* < 0.001), and 2.0 (*p* < 0.001) years, respectively, compared with the age-matched controls who had parents died at the age of average life expectancy. No differences existed in the ages of onset for age-related but non-cardiovascular diseases, such as cancer, cataract, or osteoporosis [18]. After 3.5 ± 1.7 years of follow-up, the risks of myocardial infarction (OR: 0.22; 95% CI: 0.05–0.92; *p* = 0.04), diabetes mellitus (OR: 0.14; 95% CI: 0.04–0.55; *p* = 0.005), and stroke (OR: 0.17; 95% CI: 0.05–0.57; *p* = 0.004) were significantly lower in centenarian offspring compared with the referent cohort. No significant differences existed in hypertension, arrhythmia, or other non-cardiovascular diseases [19].

The protective effects of paternal longevity on cardiovascular diseases were shown not only in centenarian offspring, but also in populations who lost a parent during childhood. Studies revealed that death of a parent during childhood affects 3% to 4% of children in the Western world [20] and may increase the blood pressure level in late adolescence or adulthood [21]. In a prospective study of Swedish men, losing a parent during childhood was associated with an increased risk of ischemic heart disease (adjusted hazard ratio (HR): 1.30; 95% CI: 1.13–1.49) during the 39-year follow-up. No difference was found for stroke (adjusted HR: 0.87; 95% CI: 0.66–1.15). The abovementioned studies were in line with our MR results, indicating that paternal longevity may causally decrease the risks of coronary heart disease and peripheral artery disease. Compared with traditional observational studies, MR analysis is less likely to be driven by confounding factors as genetic variants are randomly allocated at conception, which resembles the random assignment of participants to experimental and control groups in a randomized controlled trial (RCT). Moreover, MR analysis overcomes reverse causality, as genetic variants are fixed regardless of the onset or progression of a disease [22]. While RCTs are in principle the best way to determine the causal association of a particular exposure, RCTs are expensive and time-consuming, especially for rare outcomes or outcomes requiring a long follow-up period to be observed. Additionally, many exposures cannot be randomly allocated for practical or ethical reasons. In that case, MR may be an alternative way to infer causal association [23]. Therefore, our results provided directly causal evidence that more aggressive support and attention are required for cardiovascular risk factors and cardiovascular disease management for populations with premature paternal death.

The mechanisms between paternal longevity and lower risks of cardiovascular diseases may be explained by the following reasons. Firstly, offspring of long-lived parents were more likely to live a healthier life when compared with the controls, such as a significantly lower body mass index (25.6 vs. 28.2, *p* < 0.05 for female; 27.2 vs. 29.4, *p* < 0.05 for male) [2], smaller proportion of obese individuals, and higher ability to walk 500 m without requiring help [24]. Parental longevity was associated with less decline in physical function in aging [25], and maintaining physical function in old age is a crucial component of healthy aging [26]. Second, paternal longevity was associated with a lower frequency of carotid plaques and decreased aortic arterial stiffness. In the SUVIMAX Vascular Study, volunteers were divided into the following three groups according to father’s age at death: ≤65 (*n* = 280), 66–80 (*n* = 421), and >80 years (*n* = 364). Compared with the subjects whose fathers deceased by 65 years, those subjects in the 66–80 years (OR: 0.68; 95% CI: 0.48–0.96) and >80 years (OR: 0.69; 95% CI: 0.49–0.98) groups had a lower risk of carotid plaques after adjusting for conventional cardiovascular risk factors (*p* for trend = 0.006). The mean common carotid arteries intima-media thickness was also higher in subjects whose father died before 65 years in univariate analysis (*p* for trend = 0.007), although the difference disappeared after multivariate adjustment (*p* for trend = 0.44). The multivariate-adjusted means of carotid-femoral pulse-wave velocity, which was used to assess aortic arterial stiffness, were 11.9 ± 0.14, 11.7 ± 0.12, and 11.0 ± 0.12 m/s (*p* for trend <0.0001), respectively, in the ≤65, 66–80, and >80 years groups. Aortic arterial stiffness was a predictor of cardiovascular events in the general population [27]. Third, children exposed to the death of a parent were more likely to experience low social support, financial problems, partner attachment difficulties, and lower educational attainment than their unexposed counterparts [28]. Long-term exposure to such social adversities may lead to lifestyle changes and alterations in endocrine, immune, vascular, and hemostatic activities, ultimately increasing the risks of cardiovascular disease [29]. Parental longevity may represent a combination of genetic, behavioral, and environmental factors that are transmitted across generations. During young adulthood, the survival advantage may be mainly explained by environmental and behavior-related factors, while at older ages, genetic factors may play an increasingly significant role [30]. In the present MR analysis, the selected SNP used as instrumental variables for parental longevity included rs429358, which is located in the APOE gene. APOE was associated with longevity and aging-related diseases [31]. Therefore, offspring may inherit genetic factors that protect against cardiovascular diseases.

Although we cannot control the genes we inherit, we can modify the behavioral and environmental factors that may result in cardiovascular diseases. Fraser et al. suggested that choices in diet, cigarette smoking, exercise, and body weight may alter life expectancy by many years [32]. Furthermore, according to the New England Centenarian Study [18], if longevity is due to a delay in cardiovascular disease instead of a general avoidance of age-related diseases, then measures focusing on preventing cardiovascular risk factors may be beneficial not only for avoiding cardiovascular disease but also for living longer in healthy condition. The causal role of paternal longevity for decreased risks of coronary heart disease and peripheral artery disease suggested that paternal longevity may be an important parameter for evaluating cardiovascular risk profiles. Populations with premature paternal death need more aggressive support and attention for cardiovascular risk factors and cardiovascular diseases.

Several limitations are deserving our attention. First, we explored the genetic effect of paternal longevity on cardiovascular diseases in predominantly European populations. Whether the results can be generalized to other populations remains to be determined. However, considering that population stratification might also affect MR estimates, the European origin can exclude the influence of population stratification bias on results. Second, we were unable to assess whether there was a non-linear trend between paternal longevity and the risks of cardiovascular diseases in the present MR analysis as individual-level data were not available. Third, the heterogeneity in the majority of clinical outcomes was substantial. Therefore, IVW under a random-effects model was adopted to mitigate the influence of heterogeneity. Moreover, weighted median and multivariable MR yielded consistent results. Fourth, differences may exist between males and females in the face of stress [33,34]. However, we had no details about gender composition in the original GWAS, which limits us to make a further analysis based on gender composition. Finally, we lacked information on the causes of paternal death, which made evaluating the risks of cardiovascular diseases based on death causes impossible.

## 5. Conclusions

In summary, our genetic analysis supported the causal role of paternal longevity for decreased risks of coronary heart disease and peripheral artery disease. While bereavement is unavoidable, understanding its health effects may help highlight the need for better monitoring and intervention of cardiovascular diseases. Future studies including different comparison populations may help us further understand the impact of paternal longevity on the risks of cardiovascular diseases.

## Figures and Tables

**Table 1:** Single-nucleotide polymorphisms used as instrumental variables in the Mendelian randomization analyses.

SNP	Chr	EA	NEA	Beta	SE	Nearby Gene	F
rs59660701	2	T	C	−0.0166	0.003	LOC105373782	30
rs13070730	3	C	T	0.0133	0.0024	FGD5	30
rs11709525	3	T	C	0.0147	0.0026	BSN	31
rs186696265	6	T	C	−0.0701	0.0099	NA	50
rs4714070	6	T	C	0.0132	0.0024	LINC02520	30
rs2179517	6	C	G	0.0138	0.0024	H2AC7/H2BC7/H3C4	33
rs1556516	9	C	G	−0.0222	0.0024	CDKN2B-AS1	86
rs10774625	12	G	A	0.0132	0.0024	ATXN2	30
rs2071382	15	C	T	0.0139	0.0024	FES	33
rs56390833	15	A	C	−0.0239	0.0026	CHRNA5	88
rs429358	19	C	T	−0.0245	0.0033	APOE	55
rs12461964	19	G	A	−0.0138	0.0024	NA	33

Chr, chromosome; EA, effect allele; NA, not avaliable; NEA, non-effect allele; SE, standard error; SNP, single-nucleotide polymorphisms.

**Table 2:** Associations between genetically predicted father’s age at death and cardiovascular diseases.

Outcomes	Weighted Median		MR-Egger		Multivariable MR	
	OR (95% CI)	*p*	OR (95% CI)	*p*	OR (95% CI)	*p*
Coronary heart disease	0.12 (0.05–0.30)	<0.001	0.02 (0–2.72)	0.156	0.16 (0.10–0.25)	<0.001
Hypertension	0.90 (0.50–1.64)	0.740	0.86 (0.45–1.63)	0.651	0.54 (0.38–0.76)	0.001
Atrial fibrillation	1.12(0.75–1.67)	0.575	0.60 (0.13–2.86)	0.540	0.77 (0.55–1.08)	0.129
Heart failure	0.46 (0.27–0.77)	0.003	0.23 (0.02–3.04)	0.297	0.47 (0.34–0.66)	<0.001
Transient ischemic attack	1.32 (0.53–3.31)	0.549	1.30 (0.44–3.85)	0.653	0.67 (0.39–1.14)	0.137
Ischemic stroke	0.74 (0.41–1.35)	0.328	0.87 (0.47–1.59)	0.658	0.76 (0.55–1.06)	0.103
Peripheral artery diseases	0.12 (0.03–0.43)	0.001	0.14 (0–9.67)	0.391	0.49 (0.26–0.93)	0.029
Cardiac death	0.59 (0.21–1.60)	0.299	1.05 (0.11–9.77)	0.970	0.38 (0.21–0.70)	0.002

CI, confidence interval; MR, Mendelian randomization; OR, odds ratio.

**Table 3:** Assessment of heterogeneity and directional pleiotropy.

Outcomes	Heterogeneity		Pleiotropy	
	Q	*p*	Intercept	*p*
Coronary heart disease	137	<0.001	0.026	0.57
Hypertension	82	<0.001	−0.044	0.16
Atrial fibrillation	29	<0.001	0.009	0.53
Heart failure	58	<0.001	0.014	0.56
Transient ischemic attack	22	0.004	−0.030	0.31
Ischemic stroke	52	<0.001	−0.061	0.06
Peripheral vascular diseases	30	<0.001	0.002	0.97
Cardiac death	9	0.358	−0.013	0.55

## Data Availability

Data available in a publicly accessible repository that does not issue DOIs. Publicly available datasets were analyzed in this study. This data can be found here: (https://gwas.mrcieu.ac.uk/datasets/) on 22 May 2022.

## Supplementary Materials

The following supporting information can be downloaded at: https://www.mdpi.com/article/10.3390/jcdd9080233/s1, Figure S1: Leave-one-out sensitivity analysis of the association of father’s age at death with eight cardiovascular diseases; Figure S2: Funnel plot of the association of father’s age at death with eight cardiovascular diseases; Table S1: Baseline characteristics of father’s age at death and cardiovascular diseases.
