# Supplementary material for: Causal Associations between Paternal Longevity and Risks of Cardiovascular Diseases
Source: J Cardiovasc Dev Dis. 2022 Jul 26;9(8):233. doi: 10.3390/jcdd9080233 (PMC9332106; doi:10.3390/jcdd9080233)
Supplement: Supplementary file 1 [file jcdd-09-00233-s001.zip › Table S1.pdf]

Table S1. Baseline characteristics of father's age at death and cardiovascular diseases

Trait	Year	Author	Population	Sample Size	n SNP	n case	n control
Father's age at death	2018	Ben Elsworth	European	341,118	9,851,867	NA	NA
Coronary heart disease	2015	Nikpay	European (~ 74%), Asian (~ 26%)	184,305	9,455,779	60,801	123,504
Hypertension	2021	-	European	218,792	16,380,466	42,857	175,935
Atrial fibrillation	2021	-	European	127,442	16,379,586	10,516	116,926
Heart failure	2020	Shah S	European	977,323	7,773,021	47,309	930,014
Transient ischemic attack	2021	-	European	214,634	16,380,437	8,835	205,799
Ischemic stroke	2018	Malik R	European	440,328	7,537,579	34,217	406,111
Peripheral artery diseases	2021	-	European	218,792	16,380,466	1,037	217,755
Cardiac death	2021	-	European	218,792	16,380,466	7,563	211,229

NA, not available; SNP, single nucleotide polymorphism
